# Real time study of grain enlargement in zirconium under room-temperature compression across the α to ω phase transition

**DOI:** 10.1038/s41598-019-51992-2

**Published:** 2019-10-31

**Authors:** Dmitry Popov, Nenad Velisavljevic, Wenjun Liu, Rostislav Hrubiak, Changyong Park, Guoyin Shen

**Affiliations:** 1 High Pressure Collaborative Access Team, X-ray Science Division, Argonne National Laboratory, Lemont, Illinois, USA; 2 Shock and Detonation Physics Group, Los Alamos National Laboratory, Los Alamos, New Mexico, USA; 3 X-ray Science Division, Argonne National Laboratory, Lemont, Illinois, USA; 4 Physics Division-Physical & Life Sciences Directorate, Lawrence Livermore National Laboratory, Livermore, California 94550 USA

**Keywords:** Phase transitions and critical phenomena, Phase transitions and critical phenomena

## Abstract

We report a synchrotron Laue diffraction study on the microstructure evolution in zirconium (Zr) as it undergoes a pressure-driven structural phase transformation, using a recently developed real time scanning x-ray microscopy technique. Time resolved characterizations of microstructure under high pressure show that Zr exhibits a grain enlargement across the α-Zr to ω-Zr structural phase transition at room-temperature, with nucleation and growth of ω-Zr crystals observed from initially a nano-crystalline aggregate of α-Zr. The observed grain enlargement is unusual since the enlargement processes typically require substantially high temperature to overcome the activation barriers for forming and moving of grain boundaries. Possible mechanisms for the grain enlargement are discussed.

## Introduction

It is well known that variation in pressure (P), like in temperature (T), can lead to significant changes in chemical, structural, mechanical, electronic, magnetic, and other material properties^1,2^. Group-IV elemental metals titanium (Ti), zirconium (Zr), and hafnium (Hf), along with their many alloys, show many desirable properties and are important in a broad range of engineering applications, including aerospace, nuclear power, and medical industry^3^. Due to their importance, the effects of high pressure on the physical behavior of these metals are some of the most broadly investigated^4^.

In some of the earliest high-pressure experiments, with coupled electrical resistance measurement as a diagnostic, it was observed that Zr undergoes a possible discontinuous first-order type structural phase transformation under room-temperature compression^5^. In 1963, using x-ray diffraction and diamond anvil cell (DAC), Jamieson^6^ confirmed that Ti, Zr, and Hf, do indeed undergo a pressure driven transformation from hexagonal close-packed (referred to as the α phase) structure at the ambient P-T condition to another hexagonal (denoted as ω) phase at high pressures. Numerous studies have since confirmed the existence of the first-order solid-solid α-ω structural phase transformation, as well as identifying another transformation to body-centered cubic (commonly referred to as β phase) structure at higher P-T conditions^7^. Over the course of last century there has been an immense amount of studies focused on determining the α-ω-β boundary at high P-T conditions, as well as investigating the role of alloying/impurities and measuring corresponding effects on the phase boundaries and transformation kinetics^8^. With the advances in high-pressure experimental platforms and diagnostic capabilities, a deeper comprehension, such as insight into the transformation mechanism(s) and other microstructural changes of Zr under high pressure conditions, has been gained recently^9,10^. It was demonstrated that mechanism of the α → ω transition in Zr substantially affects texture development under high pressure. For instance, the phase transition induced texture can be explained by orientational variants^10^. The orientation relations between α- and ω-Zr, derived from texture, support the Silcock/Rabinkim direct transformation mechanism versus the possibility of transition through the intermediate β-phase^11^.

Our current work is motivated by the fact that, in some of the high-pressure investigations of structural phase transitions, there has been experimental evidence suggesting that Zr, as well as some other materials, exhibit an unusual grain enlargement induced by pressure during the structural phase transformation without increase of temperature^12–15^. However, the evidence of grain enlargement in the resulting high-pressure product phase so far has been suggested essentially based on the relative change to a “spotty” x-ray diffraction pattern from the “smooth” diffraction pattern collected from the starting parent phase heaving finer grain size^12–15^. Although the relative change in appearance of diffraction pattern supports the grain size increase qualitatively, it may not be conclusive by itself. Further quantitative measurements of grain morphology during the grain enlargement process in zirconium have not been reported.

Grain enlargement is promoted typically by the thermal energy to overcome activation barriers for diffusion, motion of dislocations, and migration of grain boundaries including newly formed ones^16–18^, so the appearance of this phenomena during the room-T compression is unexpected. To better understand the structural phase transformation process and the corresponding microstructural evolution, a spatially resolved and time resolved characterization of the microstructure during the α → ω transition is required. In principle, microstructural information can be obtained by using high-pressure single-crystal or multi-grain x-ray diffraction experiments with monochromatic x-ray beam and an area detector. However, single/multi-grain measurements with a monochromatic x-ray beam lack the proper time-resolution to collect the necessary reflections and identify the grain enlargement with relevant temporal and spatial resolution, and this is mainly due to the experimental constraints and requirement for prolonged sample rotation to satisfy the Bragg condition. Conversely, the Laue approach uses polychromatic beam, which does not require rotation of the sample and, therefore, provides multiple orders of magnitude improvements in temporal resolution for determining deformation, mosaicity, strains, and relative grain size change^2,12,19–22^. In this study, we have applied a recently developed real time scanning x-ray diffraction microscopy (SXDM) technique based on synchrotron Laue diffraction^21,22^ to monitor the microstructure during the α → ω phase transition of Zr. We report the first direct observation of grain enlargement from an initial nano-grained high purity Zr sample as it transforms from the ambient-pressure α phase into the high-pressure ω phase at room temperature.

## Results

**Figure 1:**
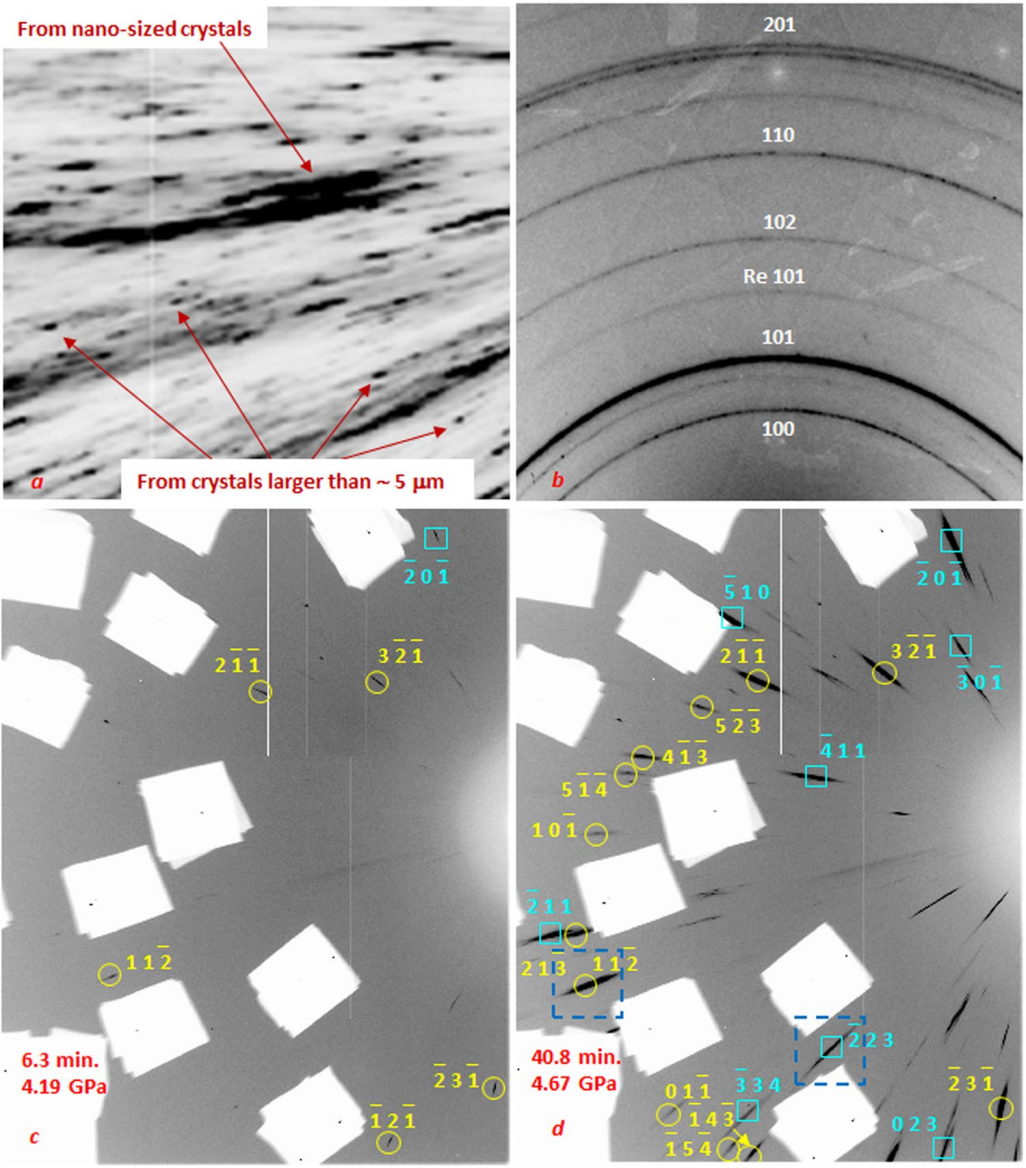
Diffraction patterns from the high purity Zr sample. (**a**) Laue image obtained with x-ray beam of ~8 × 8 μm^2^ size collected from the starting bulk sample; the diffraction pattern shows that the initial sample consists of textured component, with a mix of nanometer size grains, as characterized by the elongated streaks, and grains of a few tens of μm in size, as indicated by much sharper diffraction spots. (**b**) Angle dispersive diffraction pattern obtained with ~0.5 × 0.5 µm^2^ sized monochromatic beam from the sample as loaded in a DAC at 0.63 GPa; indices of the strongest diffraction lines of α-Zr are shown along with the strongest line from Rhenium gasket. (**c**,**d**) Laue images collected with ~8 × 8 μm^2^ sized X-ray beam as the pressure was increased and the α → ω transition began. Shown are snapshot images collected at 6.3 min (**c**) and 40.8 min (**d**), while all of the compiled time-resolved images can be seen as a movie (Supplementary Movie 1). The small pressure increase observed over this time frame can be attributed to expected relaxation of gasket, as well as the result of relative change in sample volume due to α → ω transition. Indices of reflections of two ω-Zr crystals, called grain 1 (yellow) and grain 2 (blue), are also indicated; the intensity scaling is the same for both images as defined in Fit2d software^27^. Blue dotted square in ***d*** shows the two reflections that we selected and used to further demonstrate grain enlargement, as can be seen in Fig. 2. (**a**,**b**) White rectangular areas appearing in the images are due to pieces of lead, which were used to mask strong Laue reflections from diamonds.

The Laue image obtained with x-ray beam of ~8 × 8 μm^2^ size shows that the initial sample was comprised of a mix of grains that were textured and varied in sizes from a nano-meter range to a few tens of micrometers (Fig. 1a). After the bulk sample was scratched on the surface and small sample pieces with sizes of ~40 μm in diameter and ~5–10 μm thicknesses were loaded in DACs for high-pressure experiments, the samples consisted of nano-sized randomly orientated grains as was indicated with monochromatic beam diffraction using 0.5 × 0.5 μm^2^ beam (Fig. 1b). Likewise, results of polychromatic beam Laue measurements, with beams of 0.5 × 0.5 μm^2^ and ~8 × 8 μm^2^, are consistent with this monochromatic beam observation and show no sharp diffraction spots, i.e. no large grains existed in the as-loaded samples.

Further observations of the grain microstructure were made over the pressure range that overlaps the transition from α to ω (i.e., 4.10 GPa to 5.01 GPa), over a time span of 1 hour. As the pressure was increased, a time sequence of 20 planar scanning X-ray diffraction microscopy (SXDM) images^22^ of the whole sample was collected using 5 μm step with a polychromatic beam of ~8 × 8 μm^2^. No Laue reflections, i.e., large grains, were observed from the α-phase through the course of compression. The fact that the α-phase remained as nano-crystalline aggregate was also confirmed using monochromatic angle dispersive diffraction (Supplementary Fig. S1). Only when the α → ω transition started, the Laue reflections from ω-Zr were observed, as evident in the collected X-ray diffraction images (Fig. 1c,d). The number and intensities of the ω-phase reflections increased gradually and continuously with time associated with the on-going transition as the pressure was gradually increased.

We used the recorded SXDM images to obtain the evolution in positions, shapes, and orientations of the newly formed grains of ω-Zr at different locations within the sample. Identification of all ω-Zr grains would be challenging, since many of the grains did not produce enough reflections for reliable indexation due to their small sizes, large mosaicity and limitations in diffraction angles constrained by the experimental setup geometry. Here, only four grains of ω-Zr were selected in our data analysis. The resulting indexed Laue diffraction patterns from two ω-Zr crystals, referred to as grain 1 and grain 2, are shown in Fig. 1c,d as an example. These two images were recorded on the same sample position and all the images collected in series with time on this position are available as a movie (Supplementary Movie 1). As can be seen in the series, the intensities of reflections from ω crystals continue to increase over a period of ~40 minutes and then slowly level off, showing the evolution of α → ω transition and subsequent grain enlargement in ω phase.

**Figure 2:**
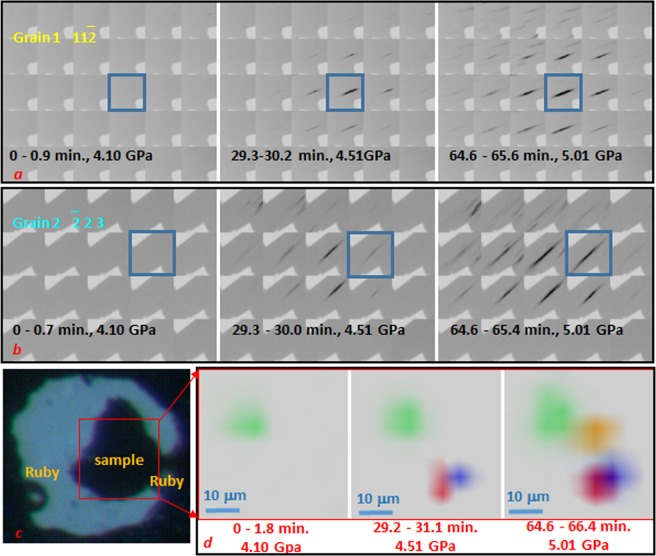
Mapping of ω-Zr grains. (**a**,**b**) composite images of selected reflections denoted by dotted squares in Fig. 1, *d* from grain 1 (**a**) and grain 2 (**b**) recorded at different time intervals after pressure increase started. For each grain, the intensity scaling is the same for all the images as defined in Fit2d software^27^. Blue squares denote x-ray images that were collected at the same position as the two patterns shown in the Fig. 1. (**c**,**d**) Step size was 5 μm. All the composite frames from grain 1 and grain 2 are also available as movies (Supplementary Movies 2 and 3). (**c**) Optical image of the sample in a DAC. (**d**) three composite SXDM images showing appearance and subsequent enlargement of ω-Zr grains as a function of time and pressure. Different color coding is used to better distinguish each grain within the sample region denoted by the red square on the optical image. Grains 1 and 2 are denoted by blue and red, respectively. All the maps are available as a movie (Supplementary Movie 4).

Further insight into the enlargement of ω-Zr grains can be gained by mapping the grains using their diffraction spots. For example, if we focus on 11 $\bar{2}$ reflection of grain 1 and $\bar{2}$ 23 reflection of grain 2, areas in diffraction images around these reflections can be combined into composite frames in the same order as diffraction patterns collected during the 2D scan (Fig. 2a,b, Supplementary Movies 2 and 3). From these composite images, we can obtain the distribution of an individual grain and its evolution over time as the experimental conditions change. As shown in the Fig. 2a,b, we can see the nucleation and subsequent enlargement of these two grains over time. The scanning x-ray diffraction images, obtained by using the XDI software^22^, further provide information on the microstructure of ω-Zr grains within the entire sample, such as the growth of the selected grains, and their orientations as well as their morphologies (Fig. 2c,d, Supplementary Movie 4).

**Figure 3:**
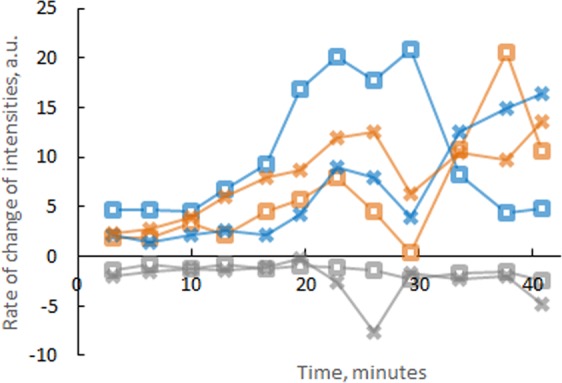
Rate of increase/decrease in average intensities of reflections with time. Blue, brown and gray plots correspond to diffraction spots from grain 1, grain 2 and from other grains in their near vicinity, respectively. Squares and crosses correspond to two neighboring areas of the sample which exhibit the strongest increase of intensities of reflections from grains 1 and 2. All such plots from the whole area around these two grains are available in Supplementary Fig. S2. Pressures right before and after these observations were 4.10 GPa and 4.67 GPa respectively.

The majority of Laue reflections exhibits positive shifts of intensities with time and pressure except some minor negative variations. This was recognized visually on the diffraction patterns and by quantitative estimates of the rates of intensity changes for selected ω-Zr crystals (Fig. 3). The minor negative shifts may be attributed to two possible explanations. (1) The reflections exhibit relatively low shifts in intensity, so they are thus more sensitive to random fluctuations in x-ray beam profile and the instability of mechanical stages used in positioning the DAC setup. (2) The growth of grains 1 and 2 may be at the expense of both α-Zr and the nucleated ω-Zr in their vicinities during a process similar to coarsening^16,18^. However, further improvements in spatial resolution and stability in the experimental setup are needed to conclusively address the coarsening process. More data of relative shifts in reflection intensities from grains 1 and 2 and those from other grains in their vicinities are presented in the Supplementary Fig. S2.

The identified ω-Zr crystals had an arbitrary orientation with respect to each other (Supplementary Fig. S3). It is interesting to note that these crystals maintained the mutual orientations as the grains continued to grow. Diffraction spots from these crystals exhibited only small shifts over time relative to the original positions on the X-ray images, indicating minor reorientations of the grains with reorientation angles less than ~1°.

**Figure 4:**
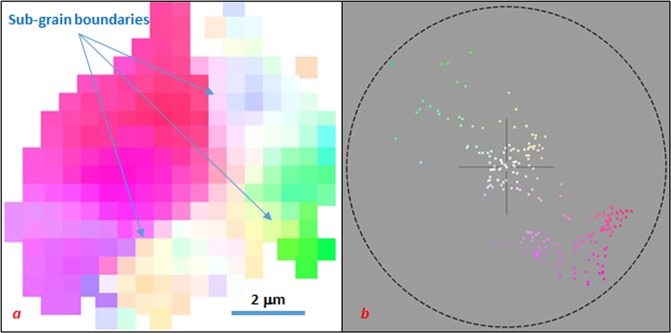
Crystal orientation map (**a**) of an ω-Zr grain generated with program LaueGo^26^. The map is colored based on calculated (001) pole figure. (**b**) The pole figure colors represent the mis-orientation of each volume element. Sub-grain structures with mis-orientation of <0.5 degrees are shown.

The grain enlargement of ω-Zr involves a notable mosaic spread, as indicated from streaky diffraction spots in Laue patterns (Figs 1 and 2). When we used a small x-ray beam (0.5 × 0.5 μm^2^) for mapping microstructure inside a large grain, it is found that the enlarged ω-Zr crystals have low-angular sub-grain boundaries (Fig. 4).

## Discussion

As shown in Fig. 3, positive shifts of reflection intensities from grains 1, 2 clearly dominate over the negative shifts of reflections from other crystals in the near vicinities. This suggests that the ω-Zr crystals were growing at expense of mainly α-phase rather than other ω-Zr grains, because, if the grain enlargement in Zr involved a process similar to normal growth^16,18^, grains 1 and 2 would grow at the cost of the other ω-Zr grains and their neighboring crystals would exhibit negative shifts of intensities comparable to the positive shifts of grains 1 and 2. However, the minor negative shifts in Fig. 3 are only randomly distributed, without clear correlations with the dominant positive shifts from grains 1 and 2. The random distribution may be a consequence of limited spatial resolution and stability in our experimental setup. Thus, further studies are required to address whether some minor negative shifts of intensities may be an indication of a process in ω-Zr similar to grain coarsening^16,18^.

It is widely known that the processes of grain enlargement, including recrystallization, recovery and grain coarsening, are driven by release of energy stored during deformation^16–18^. As the studied samples were deformed before they were subjected to high pressure, the grain enlargement in Zr may be attributed to the similar driving force. In our case, however, it seems likely that the α → ω phase transformation provides more effective route to release the stored energy than room temperature thermal diffusion does. The process necessarily combines the nucleation that does not require an atomic diffusion, i.e., a displacive (martensitic) phase transformation mechanism as known for this material, and the growth that utilizes the energy stored in the surrounding parent phases in the form of deformation. With deformation as the driving force, the rate of growth seems apparently faster than that of nucleation in our observation as a faster growth rate will cause grain enlargement.

The color-coded maps, as shown in Fig. 2d and Supplementary Movie 4, present the nucleation point of the ω transformation and subsequent grain enlargement with time. Likewise, the maps exemplify the locations and morphologies of four selected large ω grains with respect to the actual Zr sample. As can be seen from the composite frames of Laue reflections (Fig. 2a,b, Supplementary Movies 2 and 3), the ω-Zr grains nucleated and grew, as was indicated by systematic decrease of intensities of their reflections from the points of nucleation towards edges of the grains. Maps of the four identified ω-Zr crystals presented in Fig. 2d and in Supplementary Movie 4 show that the newly formed ω-Zr crystals nucleated separately from each other, with random orientations, at different times and pressures, and then grew independently, i.e., process was highly localized in both time and space.

The parental phase consists of nano-sized crystals heaving random orientation as was indicated by diffraction patterns obtained with X-ray monochromatic beam heaving size of ~0.5 × 0.5 µm^2^ (Fig. 1b) At the same time the newly formed crystals of ω-Zr have size in a range of microns as they produced strong Laue reflections with ~8 × 8 μm^2^ sized X-ray beam (Fig. 1d). These observations indicate that the ω-Zr crystals are surrounded by randomly oriented nano-szied crystals of the parental α-Zr which supports a grain enlargement mechanism by formation and motion of high-angular grain boundaries (with greater than 10–15° mis-orientation) between the ω-Zr crystals and surrounding materials^16^. This seems similar to the recrystallization process that would take place at elevated temperatures, while it distinguishes from a recovery process in which stored energy of dislocations is released without movement of the high angular grain boundaries^16,17^, because, in the latter case, intensities of reflections on the composite frames would increase essentially simultaneously over a grain area. In case of recovery Laue reflections from ω-Zr would rather exhibit gradual change from relatively broaden towards more sharp reflections while the observed diffraction spots stay at the same level of broadening during the grain enlargement process indicating that there is no substantial gradual texture development involved in the enlargement process.

Getting of comprehensive understanding of the mechanism and driving force of the grain enlargement process requires further experimental investigations. Application of the real time Laue diffraction with X-ray beam heaving size less than 100 nm^23^ seems to be a very promising approach for the future studies because it can provide direct information on the interaction between the nano-crystals of α-Zr and the growing crystals of ω-Zr. With enough spatial and temporal resolutions, one would be able to recover the sample specifically during the grain enlargement process.

## Method

Zr sample used in our study is of high purity, with concentrations of C, N, O, V, Al, Fe and Hf impurities less than 50 wt ppm for each element. The source of the Zr sample is the same as in these references^14,24^. The initial Zr sample was deformed prior to applying of high pressure by scratching off a small foil section of about ~10 μm thick and ~40 μm in diameter from the initial 1 cm × 1 cm × 0.5 cm disk.

DACs had total openings of ~50° on both sides and 300 μm diameter culets. Helium or neon was used as pressure transmitting medium to provide quasi-hydrostatic sample conditions up to the highest pressures reached in these experiments. Rhenium gasket was pre-indented down to a thickness of 40–50 μm and the diameter of the gasket hole after gas loading was ~70 μm. Pressure was measured with a ruby fluorescence system^25^ and pressure increase was controlled remotely using the membrane system available at the beamline.

Real time Laue diffraction measurements were conducted in transmission geometry using experimental setup at 16-BM-B beamline at the Advanced Photon Source^21^. X-ray beam was focused to 8 × 8 μm^2^ using KB-mirrors and the energy range from 10 to 70 keV was used. Mutually perpendicular translational stages were implemented to collect 2D translational scans with step of 5 μm across the sample using a Perkin Elmer area detector. Reflections from the identified grains were indexed using a custom software (see Supplementary information for details). Scanning x-ray diffraction microscopy (SXDM) images were generated using the XDI software^22^.

Measurements with x-ray polychromatic beam focused down to ~0.5 × 0.5 μm^2^ (beam profile is available in Supplementary Fig. S4) have been conducted before and after the grain enlargement process at 34-ID-E beamline of the Advanced Photon Source using energy range of 7–30 keV. Translational scans of Laue images with steps of 0.5 μm have been collected across the sample using MAR CCD area detector tilted by 45° with respect to the X-ray beam at 0.63 GPa and 5.16 GPa. The latter scan was repeated two times in order to check whether it provided reproducible data which in turn was an indication that the grain enlargement process was over and the observed variations of microstructure across the sample were due to its spatial inhomogeneity, not due to changes in time. Crystal orientation maps of an ω-Zr grain have been generated using program LaueGo^26^. Orientation of the grain was predefined using custom software (see Supplementary information). Monochromatic beam, exchangeable with the polychromatic beam, having energy of 20 keV have been used to collect diffraction images on selected points within the sample as well.

## Supplementary information

Supplementary information

Supplementary movie 1

Supplementary movie 2

Supplementary movie 3

Supplementary movie 4

## Data Availability

The data generated or analyzed during this study are available from the corresponding author on reasonable request.
