# Supplementary material for: Real time study of grain enlargement in zirconium under room-temperature compression across the α to ω phase transition
Source: Sci Rep. 2019 Oct 31;9:15712. doi: 10.1038/s41598-019-51992-2 (PMC6823495; doi:10.1038/s41598-019-51992-2)
Supplement: Supplementary file 1 — Supplementary information [file 41598_2019_51992_MOESM1_ESM.pdf]

# Supplementary information.

## Real time study of grain enlargement in zirconium under room-temperature compression across the α to ω phase transition.

D. Popov*[1], N. Velisavljevic[2], W. Liu[3], R. Hrubiak[1], C. Park[1], G. Shen[1]

[1] High Pressure Collaborative Access Team, X-ray Science Division, Argonne National Laboratory

[2] Shock and Detonation Physics Group, Los Alamos National Laboratory

[3] X-ray Science Division, Argonne National Laboratory

## Indexing of Laue reflections.

Single-crystals of ω-Zr have been identified by indexing of their Laue reflections. Software PolyLaue was developed in Python for this purpose. As the Laue images contained reflections from multiple single-crystals present in X-ray beam simultaneously, two reflections which were presumably from the same crystal were selected first in order to find crystal orientation using widely known algorithm [1]. Typically, these reflections belonged to a clear 'zone arc' which was an indication they were indeed from the same crystal. As step size of the 2D translational scans was smaller than full X-ray beam size, diffraction spots from the same crystal typically were present on multiple X-ray images; frame with the highest intensities of the selected two reflections was chosen for further data analysis. Finding of crystal orientation required knowledge of indices of the selected reflections. As they were not available a software routine to test all possible combinations of indices was developed to predict possible orientation matrices which in turn were used to attempt indexing of all other reflections present on the chosen X-ray image. The largest possible deviation limit of 0.2° between predicted and observed orientations of diffracted beams of reflections was implemented for the indexation procedure. Ranges of all possible indices have been calculated from the 2θ-angles of the selected pair of reflections and from the highest limit of X-ray energy which was defined by the tilt of KB-mirrors with respect to the incident X-ray beam. Orientation matrix provided the highest number of indexed reflections was assumed to be the correct one, if all other possible orientations provided substantially smaller number of indexed reflections. Indexation of Laue data was done using unit cell parameters of ω-Zr which were obtained with the monochromatic beam right after the Laue data were collected. Positions of Laue diffraction spots on the X-ray images have been determined using peak search function of program Fit2d [2].

Results of indexation with PolyLaue were successfully repeated using program LaueGo [3] which is widely implemented to analyze Laue data. LaueGo could not be used to analyze the data from Zr directly because this software is not efficient when reflections from too many multiple crystals are present on the same X-ray images. After diffraction spots from a separate single-crystal were identified with PolyLaue, LaueGo was

implemented to index those reflections as well. Orientation matrix of ω-Zr crystal which was selected for mapping of orientation with LaueGo (Fig. 4 of main text) was also predefined with PolyLaue.

## References


1. Chung, J.-S. & Ice, J.-S. Automated indexing for texture and strain measurement with broad-bandpass x-ray microbeams. *Journal of Applied Physics* **86**(9), 5249-5255 (1999).
2. Hammersley, A.P., Svensson, S.O., Hanfland, M., Fitch, A.N., Hausermann, D. Two-dimensional detector software: From real detector to idealized image or two-theta scan. *High Pressure Research* **14**, 235-248 (1996).
3. Tischler, J.Z. Reconstructing 2D and 3D X-ray orientation maps from white-beam Laue in Strain and Dislocation Gradients from Diffraction (ed. Ice, G. E. & Barabash, R.) Ch. 10 (Imperial College Press, London, 2014).


## Movie legends

**Movie 1.** Laue images collected with ~8 × 8 $\mu m^2$ sized X-ray beam. The movie shows collection of images from one particular sample position that was obtained during pressure increase. Indices of reflections of two ω-Zr crystals, indicated by different colors, are the same as for the two grains shown in Fig. 1, *c, d* of the main text. The relative intensity scaling was kept the same in all images as defined in Fit2d program [2].

**Movie 2.** Composite images of $(11\bar{2})$ reflections from grain 1 (Fig. 1, *c, d* of main text) recorded at different time intervals after pressure increase started (shown on each composite frame); the intensity scaling is the same for all the images as defined in Fit2d software [2]; blue squares denote X-ray images collected at the same position as patterns on Fig. 1, *c, d* of main text.

**Movie 3.** Composite images of $(\bar{2}23)$ reflections from grain 2 (Fig. 1, *c, d* of main text) recorded at different time intervals after pressure increase started (shown on each composite frame); the intensity scaling is the same for all the images as defined in Fit2d software [2]; blue squares denote X-ray images collected at the same position as patterns on Fig. 1, *c, d* of main text.

**Movie 4.** Maps of four ω-Zr grains obtained at different time intervals after increase of pressure started (shown below each map); each grain is denoted by the same color as in Fig. 2, *d* of main text. Grains 1 and 2 are denoted by blue and red colors respectively. Some variations of shape of the grains are due to random fluctuations of X-ray beam and some mechanical instability of the sample stage.

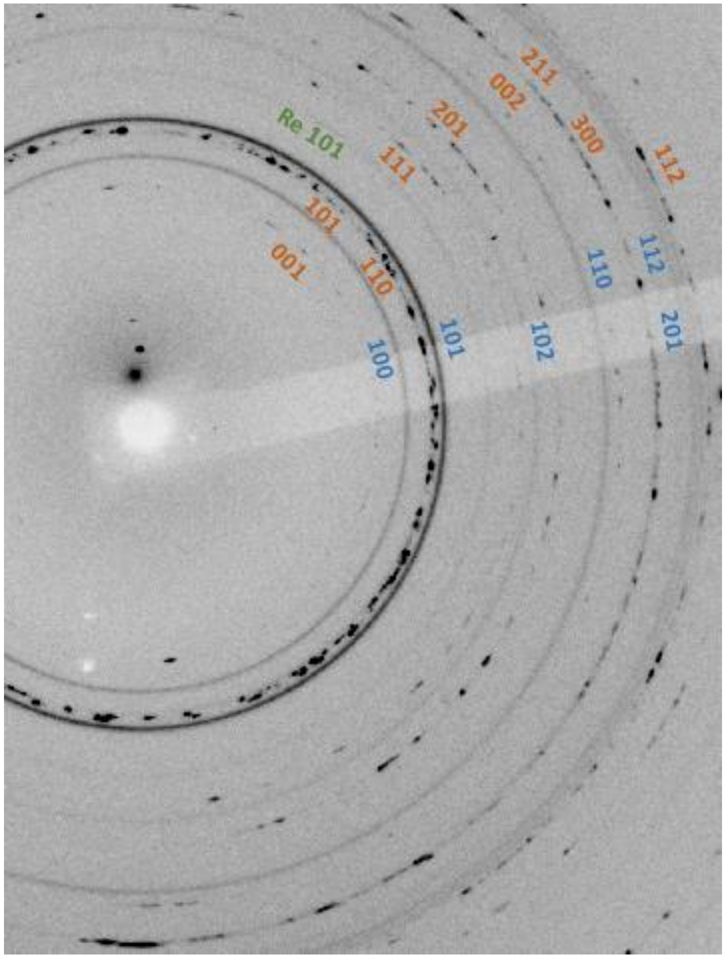


**Figure S1**. Diffraction image from a mix of α-Zr, characterized by continues lines, and from larger grains of ω-Zr produced the spotty diffractions lines. The pattern was collected at pressure of 5.7GPa at 16BM-D beamline of the Advanced Photon Source. X-ray beam was focused down to 5$\mu m^2$. Indices of diffraction lines are shown in brown and blue for ω- and α-Zr, respectively. Strongest diffraction line from Re gasket is also present and denoted by green color.

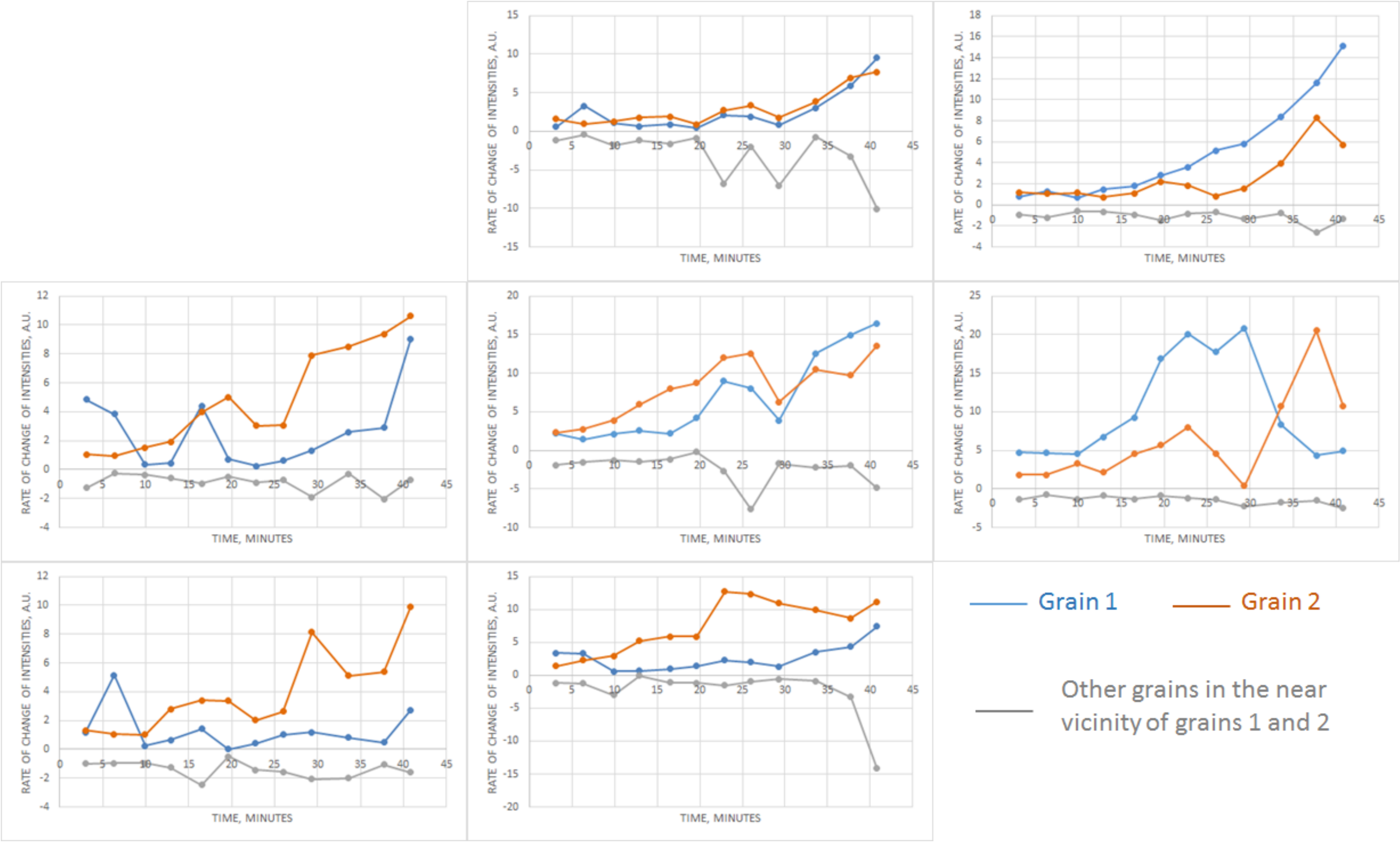


**Figure S2.** Rate of increase/decrease in average intensities of reflections with time. Blue, brown and gray plots correspond to diffraction spots from grain 1, grain 2 and from other grains in the near vicinity, respectively. Pressures right before and after these observations were 4.10 GPa and 4.67 GPa respectively. The plots are arranged in the same order as relative positions within the sample on which the data were collected.

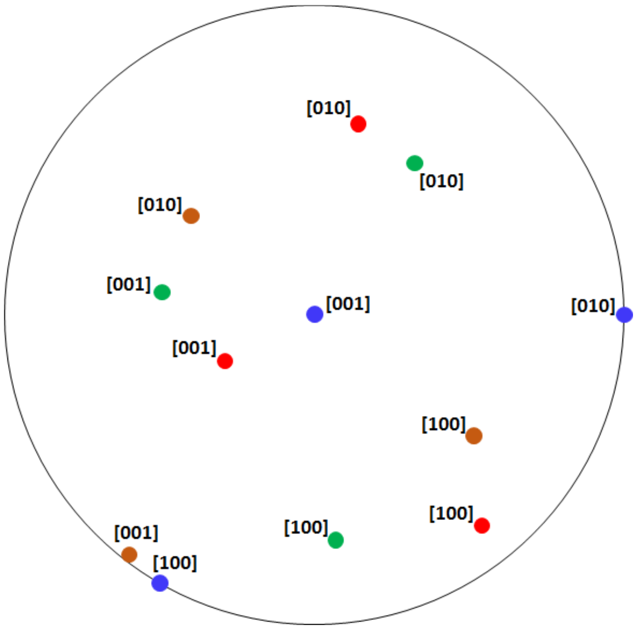


**Figure S3.** Stereographic projections of basic vectors of four identified ω-Zr crystals. Basic lattice vectors from each grain are denoted by the same color as used in Fig. 2, *d* of main text.

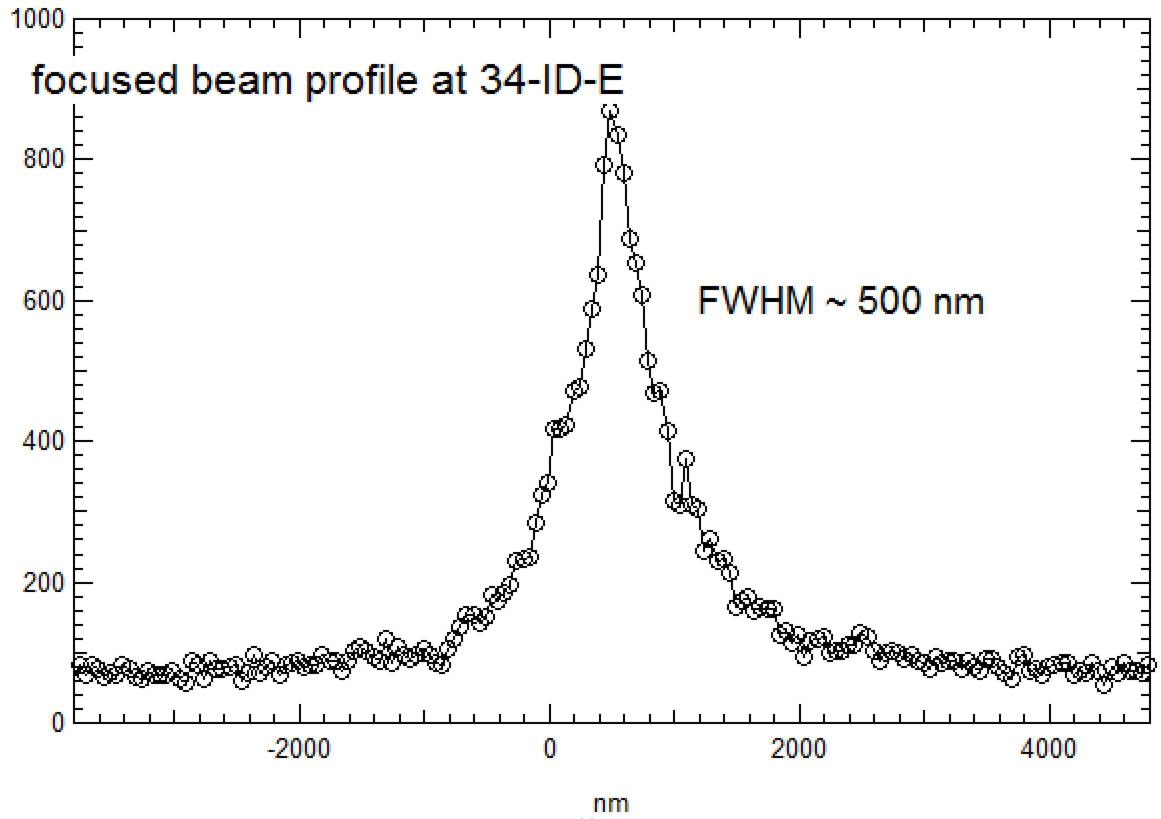


**Figure S4.** Beam profile at sample position available at beamline 34-ID-E of Advanced Photon Source.
